# Stable Sulfuric Vapor Transport and Liquid Sulfur Growth on Transition Metal Dichalcogenides

**DOI:** 10.1021/acs.cgd.2c01318

**Published:** 2023-03-21

**Authors:** Dmitriy A. Chareev, Md Ezaz Hasan Khan, Debjani Karmakar, Aleksey N. Nekrasov, Maximilian S. Nickolsky, Olle Eriksson, Anna Delin, Alexander N. Vasiliev, Mahmoud Abdel-Hafiez

**Affiliations:** † Institute of Experimental Mineralogy (IEM RAS), 142432 Chernogolovka, Moscow Region, Russia; ‡ Kazan Federal University, 18 Kremlyovskaya St., 420008 Kazan, Russia; § Ural Federal University, 620002 Ekaterinburg, Russia; ∥ University of Doha for Science and Technology, 24449 Doha, P.O. Box 24449, Qatar; ⊥ Department of Physics and Astronomy, Uppsala University, Box 516, SE-75120 Uppsala, Sweden; # Institute of Geology of Ore Deposits (IGEM RAS), 35, Staromonetnyi per., 119017 Moscow, Russia; ∇ School of Science and Technology, Örebro University, SE-701 82 Örebro, Sweden; ○ Department of Applied Physics, KTH Royal Institute of Technology, SE-106 91 Stockholm, Sweden; ◆ Swedish e-Science Research Center, KTH Royal Institute of Technology, SE-10044 Stockholm, Sweden; ¶ Lomonosov Moscow State University, 119991 Moscow, Russia; ⋈ National University of Science and Technology “MISiS”, 119049 Moscow, Russia

## Abstract

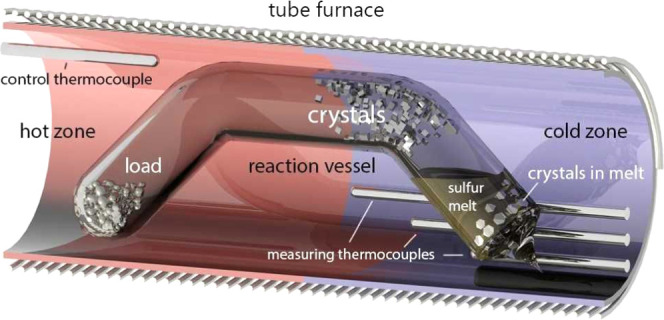

Transition metal dichalcogenides (TMDs) are an emergent class of low-dimensional materials with growing applications in the field of nanoelectronics. However, efficient methods for synthesizing large monocrystals of these systems are still lacking. Here, we describe an efficient synthetic route for a large number of TMDs that were obtained in quartz glass ampoules by sulfuric vapor transport and liquid sulfur. Unlike the sublimation technique, the metal enters the gas phase in the form of molecules, hence containing a greater amount of sulfur than the growing crystal. We have investigated the physical properties for a selection of these crystals and compared them to state-of-the-art findings reported in the literature. The acquired electronic properties features demonstrate the overall high quality of single crystals grown in this work as exemplified by CoS_2_, ReS_2_, NbS_2_, and TaS_2_. This new approach to synthesize high-quality TMD single crystals can alleviate many material quality concerns and is suitable for emerging electronic devices.

## Abstract 2

Reaction vessel in tube furnace obtaining crystals in quartz glass ampoules by chemical vapor transport technique with sulfur vapors as the transport agent. The vessel was placed into the furnace so that the left part containing the sulfide usually had a temperature of 800−850 °C and the right part with the liquid sulfur had a temperature of 550−600 °C. The 550 °C temperature of the cooler end provided sulfur fugacity sufficient for substance transport but not enough to destroy the quartz glass vessel.

Two-dimensional (2D) transition metal dichalcogenides are an emergent class of materials with growing applications in the field of nanoelectronics.^1^ Some examples of their use are in heterostructures and monolayers based on MoS_2_ and WS_2_ as transistors, MoS_2_ and MoTe_2_ as phototransistors, WS_2_, SnS_2_, and TiS_2_ as power sources, and MoS_2_, MoSe_2_, and SnS_2_ as catalysts for electrochemical water decomposition. Substances containing more sulfur, for example, trisulfides, can also have a similar layered and reduced dimensional structure. Most transition metal chalcogenides melt incongruently.^2^ Therefore, it is difficult to obtain single crystals of these substances by melt techniques such as the Bridgman or Czochralski method. Usually, crystals of these substances are obtained by the chemical vapor transport technique and less frequently by the flux technique, whereas slow cooling of the chalcogenide melts^2−5^ is the preferred method. For the vapor transport technique, most often halogens and their compounds are used as transport agents. In this case, there is a possibility of halogen incorporation into the crystal structure of the growing crystal. Hence, for the growth of diselenide crystals free from impurities of other elements, some works use selenium vapors instead as the transport agent. Transport of some dichalcogenides was also studied^2^ without the presence of free chalcogens at high temperatures. ReS_2_ crystals were grown from the Re_1_S_2.01_ charge in 900→800 °C gradient by Schäfer in several days, as reported in the article by Wehmeier et al.^3^^3^ Therein, it was assumed that sulfur fugacity was minimal while it should be determined by Re/ReS_2_ equilibrium and be significant at the temperature of synthesis. On the other hand, in other articles, Schäfer reported on the possibility of using sulfur vapors as a transport agent.^4^ The work described the growth of crystals of several sulfides including TiS_2_, V_1+δ_S_2_, NbS_2_, TaS_3_, TaS_2_, MoS_2_, WS_2_, FeS, CoS_2_, NiS_2_, PdS, and PtS, usually in temperature gradient 800 → 700 °C. Large thin transition metal dichalcogenide (TMD) crystals were grown in the excess of sulfur in 1050 → 950 °C gradient.^5^ Disulfide and diselenide crystals were obtained by transport in sulfur vapors with the pressure of 9 atmospheres.^5,6^ Previous results have shown that it was possible to obtain disulfide and diselenide crystals but not ditellurides.^7−11^ In our previous work,^12^ we noted that when evaporating selenium or tellurium from the metal and Se (Te) melt at 850–650 °C, only selenium or tellurium evaporated. On the other hand, when sulfur is added to the system, the metal could also evaporate forming mixed dichalcogenide crystals in the cooler part, for example, in Nb(Se,S)_2_. Therefore, the motivation of this work is to study the transport of transition metals and the formation of sulfide crystals in sulfur vapors and determine the optimal temperature profile for metal transport in sulfur vapors. The new insights reported in this study allow the design and synthesis of high-quality single crystals of transition metal dichalcogenides, opening the door for high-precision studies of the properties of these systems as well as new types of nanoelectronic applications.

## Results, Discussion, and Methods

**Figure 1:**
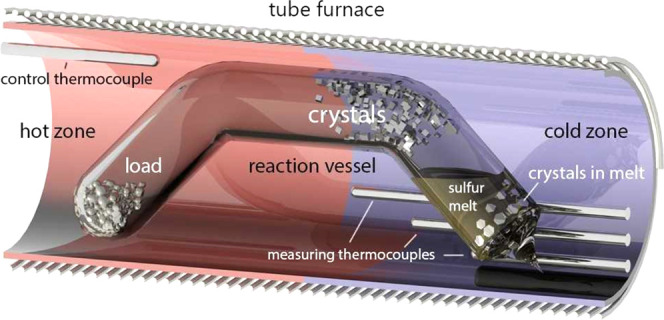
Schematic picture of the reaction vessel (ampoule). The reaction vessel in the tube furnace obtaining crystals in quartz glass ampoules by the chemical vapor transport technique with sulfur vapors as the transport agent. The vessel was placed into the furnace so that the left part containing the sulfide usually had a temperature of 800–850 °C and the right part with the liquid sulfur had a temperature of about 550–600 °C. The 550 °C temperature of the cooler end provided sulfur fugacity sufficient for substance transport but not enough to destroy the quartz glass vessel.

Quartz glass ampoules containing elementary liquid sulfur that are heated to high temperatures are extremely dangerous and unstable. The danger is represented by hot fragments of quartz glass and fumes of sulfur. All manipulations with reaction vessels were carried out with the protection of hands, face, and respiratory organs. Sulfur (Labtex 99.9%) and metals with a purity of no less than 99.9% were used as the reagents. We put metals, not the corresponding sulfides, since we believed that the process of transition of the metal to sulfide at 850 °C took no more than a few hours. Only the transport of those metals whose sulfides did not sublimate at 800–850 °C was studied. Therefore, the transport of silicon, germanium, cadmium, zinc, tin, and mercury was not studied because the crystals of their sulfides are often formed during the synthesis from elements. The reaction vessel (ampoule) was made up of quartz glass tubes with a diameter of 12 mm and a wall thickness of 2 mm (Figure 1). The tube had two bends which were previously made by the oxygen torch. This shape of the reaction vessel fixed the position of the sulfide charge and liquid sulfur. The size and shape of these bends are not important; it is important that they ensure the fixation of the load and liquid sulfur.

About 100–200 mg of metal and 1–2 g of elementary sulfur were put into the ampoules. Sulfur was taken in excess, necessary for the formation of an invariant two-phase association “liquid sulfur + gaseous sulfur”. Charged ampoules were evacuated and sealed in the flame of the oxygen torch. The total length of the vessels was 180–200 mm. The vessels were placed into the furnace so that the left part containing the sulfide usually had a temperature of 800–850 °C and the right part with the liquid sulfur had a temperature of about 550–600 °C. The 550 °C temperature of the cooler end provided sulfur fugacity sufficient for substance transport but not enough to destroy the quartz glass vessel. The highest relative safe temperature of the sulfur part is ∼640 °C at which the pressure reaches 10 atmospheres.^13^ This reaction vessel resembles the ones for the three-zone technique described in Schäfer’s works. The time scale of the crystal growth ranges from 1 to 4 months.

**Figure 2:**
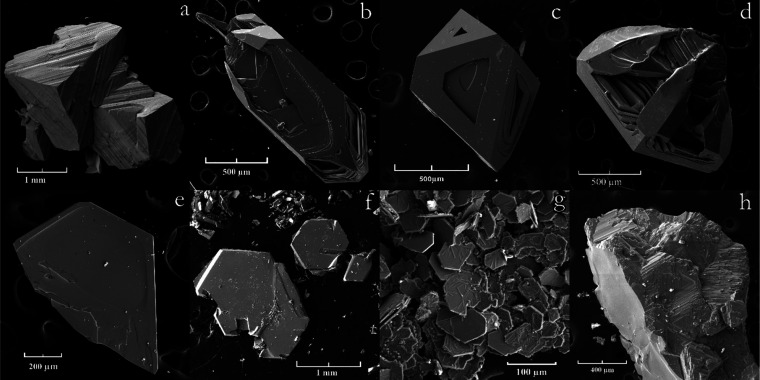
Electron microscope image of crystals. NbS_2_ (a), FeS_2_ (b), CoS_2_ (c), NiS_2_ (d), Cr_2_S_3_ (e), TiS_2_ (f), V_1+*x*_S_2_ (g), and In_2_S_3_ (h).

**Figure 3:**
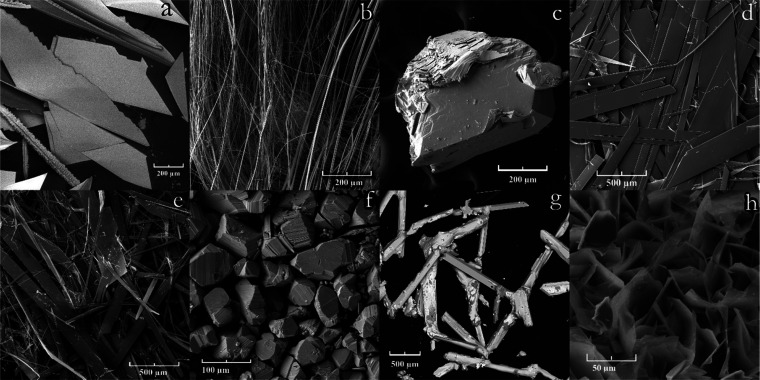
Electron microscope image of crystals. MgS (a), TaS_3_ (b), TaS_2_ (c), ZrS_3_ (d), HfS_3_ (e), PtS_2_ (f), PdS (g), and MoS_2_ (h).

All of the experiments conducted to obtain crystals by the vapor transport technique with gaseous sulfur are summarized in Table 1. The table shows the temperature range of coexistence with sulfur, the temperatures of both the hot and the cold ends of the reaction vessel, the approximate growth temperature, the time of the synthesis, the size of the crystals, and the transported amount of substance. In the case of iron, nickel, niobium, and tantalum, complete or almost complete transport of the substance up to 100 mg of metal in 2–4 months through the cross-section of 50 mm^2^ was observed. In this way, NbS_2_ (Figure 2a),^14,15^ FeS_2_ (Figure 2b), CoS_2_ (Figure 2c), NiS_2_ (Figure 2d), Cr_2_S_3_ (Figure 2e), and TiS_2_ (Figure 2f) crystals of size up to 2 mm, agglomerates of small crystals of V_1+*x*_S_2_ (Figure 2g) and In_2_S_3_ (Figure 2h), and transparent plates MgS (Figure 3a) were obtained. During the transport of tantalum in close proximity to the sulfur source, one-dimensional TaS_3_ crystals were found up to 30 mm in length and about 1 mm thick (Figure 3b). TaS_2_ crystals were located in a slightly more high-temperature part (Figure 3c). The temperature of the initial crystallization of TaS_2_ can be estimated as 675 (50) °C and that of TaS_3_ as 625 (50) °C. Similarly, red-orange ZrS_3_ (Figure 3d) and HfS_3_ crystals (Figure 3e) were obtained in a ribbon shape 10 mm long and up to 0.5 mm wide. In the experiment with platinum, 50 mg of PtS_2_ crystals were obtained in 2 months. The crystals were isometric, a few tens of micrometers in size with a well-defined layered structure (Figure 3f). Whiskers in similar conditions of PdS were formed (Figure 3g). Growing PdS_2_ crystals at a similar temperature is likely impeded due to their low temperature of stability.

**Table 1:** Parameters of Experiments on Sulfide Growth: Temperature Range of Coexistence with Sulfur, Temperatures of Both Hot and Cold Ends of the Reaction Vessel, Approximate Growth Temperature, Time of Synthesis, Size of Crystals, and Carry Amount

crystals, crystal structure	temperature range of coexistence with sulfur (°C)	temperature of hot end (of evaporation) (°C)	temperature of cold end (of condensation) (°C)	growth temperature (°C)	synthesis time (days)	crystal size	carry amount	figure
NbS_2_	n/d	850	550	∼700–600	120	1–2 mm	full	Figure 2a
NbS_3_	n/d	850	550	∼600	120	below resolution limits		
PdS	n/d	827	551	∼700–600	70	2–3 mm × 100 μm	full	Figure 3g
PdS_2_	n/d			551 (in liquid S)		2–3 mm		Figure 4d
PtS_2_	n/d	850	550	∼700–600	60	50–100 μm	low	Figure 3f
TiS_2_	>632	820	566	∼700–600	93	1 mm	full	Figure 2f
TiS_3_	<632			566 (in liquid S)		1 mm		Figure 4e
Bi_2_S_3_	113–775	620	540	∼700–600	60	3 mm × 100 μm	medium	Figure 4c
ReS_2_	n/d	820	566	∼600	93	shapeless agglomerates	low	Figure 4a
TaS_2_	n/d	820	566	n/d	93	1 mm	full	Figure 3c
TaS_3_	n/d					2–3 mm × 1 μm		Figure 3b
ZrS_3_	<700	827	551	∼700–600	70	10 mm × 0.5 mm	medium	Figure 3d
HfS_3_	n/d	827	551	∼700–600	70	10 mm × 0.5 mm	medium	Figure 3e
MoS_2_	115–1750	820	566	∼700–600	93	50 μm × 1 μm	low	Figure 3h
WS_2_ (black)	<400	820	566	n/d	93	50 μm × 1 μm	low	
WS_2_ (silver)	>400					50 μm × 1 μm		
FeS_2_ (py str.)	450–743	827	551	∼700–600	70	2 mm	medium	Figure 2b
CoS_2_	115–950	827	551	∼700–600	70	1 mm	low	Figure 2c
NiS_2_	115–998	827	551	+551 (in liquid S)	70	2 mm	medium	Figure 2d
MnS	<1653	800	572	572 (in liquid S)	120	1 mm + agglomerates of 40 μm crystals	full	Figure 4f
								Figure 4g
V_1+*x*_S_2_	n/d	800	572	∼700–600	120	agglomerates of 100 μm crystals	full	Figure 2g
Cr_2_S_3_	<1565	815	560	∼700–600	115	1 mm	full	Figure 2e
In_2_S_3_	1090	815	560	∼700–600	115	agglomerates of 100 μm	full	Figure 2h
MgS	n/d	815	560	∼600	115	1 mm plates	low	Figure 3a
Au + ZnS	1718	815	560	∼700–600	115	agglomerates of 100 μm	medium	Figure 4b
RuS_2_	n/d	800	572		120		absent	
Rh_2_S_3_ or RhS_3_	n/d	800	572		120		absent	
OsS_2_	n/d	800	572		120		absent	

**Figure 4:**
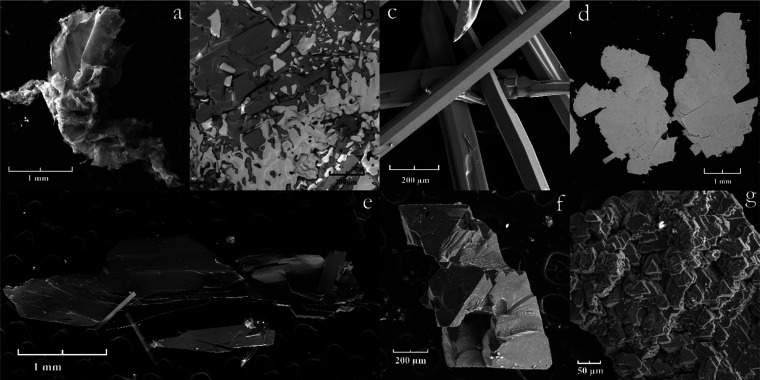
Electron microscope image of crystals. ReS_2_ (a), Au (white) and ZnS (b), Bi_2_S_3_ (c), PdS_2_ (d), TiS_3_ (e), MnS (f), and MnS agglomerates (g).

Transport of rhenium, molybdenum, and tungsten was also observed in similar temperature conditions but significantly less in volume. MoS_2_ (Figure 3h) and WS_2_ crystals a few tens of micrometers in size and ReS_2_ crystals without a well-defined habit (Figure 4a) were obtained. Tungsten disulfide crystals located right next to the sulfur source were silver in color. Black and silver areas had well-defined borders, and therefore, it can be assumed that two crystal modifications of WS_2_ were obtained. The possibility of transferring gold is shown in the standard temperature regime during the recrystallization of the ZnS + Au charge (Figure 4b). In the case of ruthenium, rhodium, and osmium, no transport was observed. Bi_2_S_3_ whiskers (Figure 4c) were obtained under the most low-temperature regime: charge temperature—620 °C and sulfur source temperature—540 °C. In the experiments to transport palladium, nickel, and titanium, some additional crystals were found in the sulfur melt, that is, in the coolest part of the reaction system. Most likely gaseous metal compounds dissolved in the liquid sulfur, diffused to the coolest part of the system, and formed the crystals there. This way NiS_2_, Cr_2_S_3_, and MgS crystals were obtained with a procedure similar to that obtained by vapor transport. In contrast, during the transport of palladium and titanium, PdS_2_ (Figure 4d) and TiS_3_ (Figure 4e) crystals were obtained instead of PdS and TiS_2_, respectively. This can be explained not by different chemical properties of liquid and gaseous sulfur, but by different temperatures of stability of the phases, see Table 1. In the case of the transport of manganese, MnS crystals (Figure 4f) and crystal agglomerates 40 μm in size (Figure 4g) were found only in liquid sulfur. To show that during the transport, the metal enters the gas phase in the form of molecules containing a greater amount of sulfur than the charge and the growing crystal, experiments were conducted without elementary sulfur. The ampoule containing the mixture of NbS_2_ and a small amount of NbS_3_ was maintained in 850 → 700 °C gradient for 2 months. The absence of transport in this experiment demonstrates that sulfur fumes are necessary for transport, which makes this technique different from the sublimation technique.

**Figure 5:**
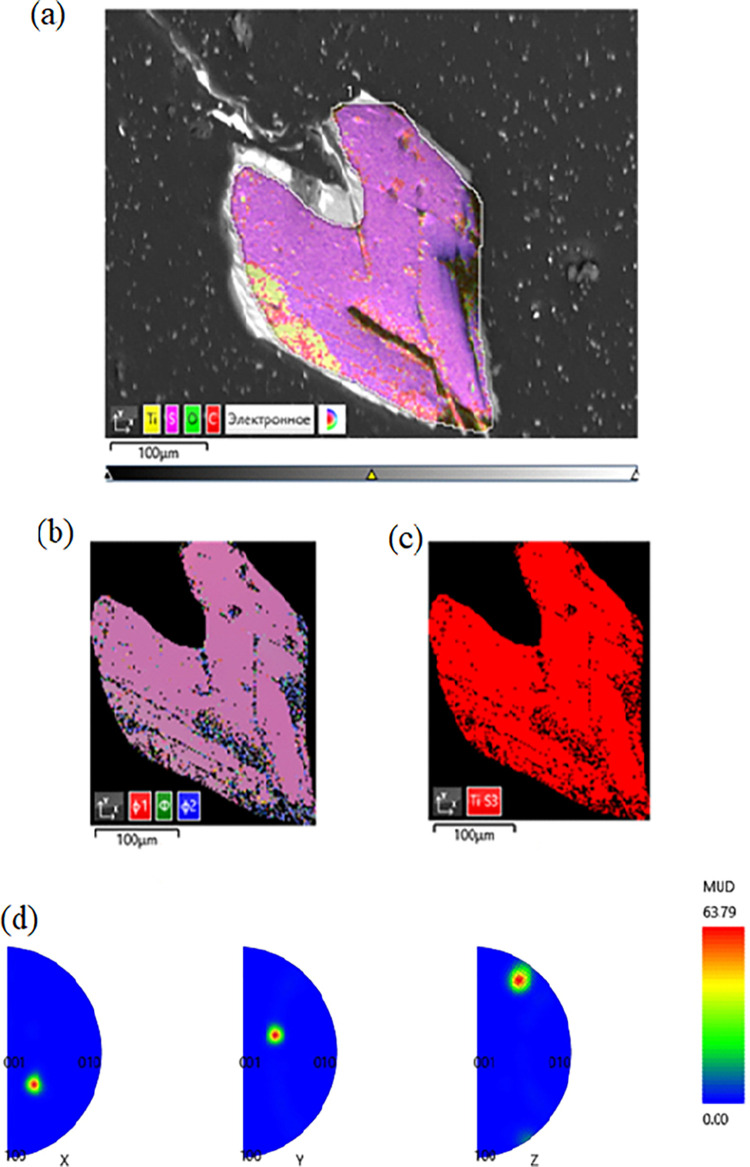
EBSD mapping of the TiS_3_ crystal. (a) Analyzed area of the crystal, (b) Euler colors, (c) phase color, and (d) inverse pole figures.

**Figure 6:**
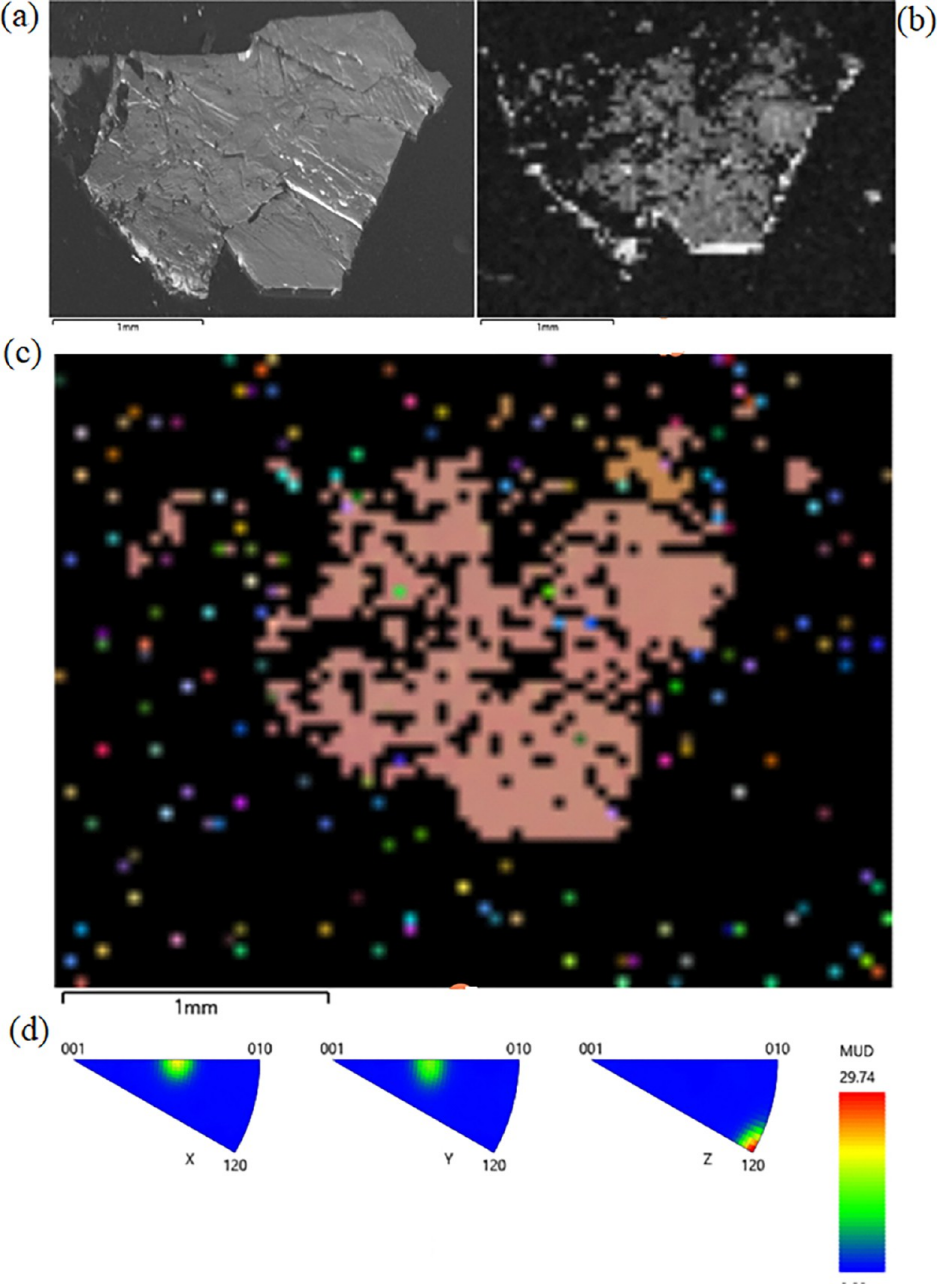
NbS_2_ EBSD mapping. (a) Analyzed crystal, (b) band contrast, (c) Euler map, and (d) inverse pole figures.

Electron backscatter diffraction (EBSD) mapping confirmed the single-crystal nature of the TiS_3_ and NbS_2_ crystals. The analyses were performed on a JEOL JSM 5610-lv electron scanning microscope equipped with an UltimMax-100 EDS detector and a Symmetry EBSD detector. The uniform Euler color, phase color, and inverse pole figures clearly demonstrate that the studied TiS_3_ crystal is a single crystal (Figure 5). The very same results were obtained with the NbS_2_ crystal. The analyzed crystal was relatively big, around 2 mm in diameter (Figure 6a). We analyzed a natural crystal face, only slightly polished, which is manifested in a poor band contrast (Figure 6b). However, the Euler map is uniform, and the inverse pole figures demonstrate that all of the analyzed points inside the crystal are oriented in one and the same direction.

## Discussion

**Figure 7:**
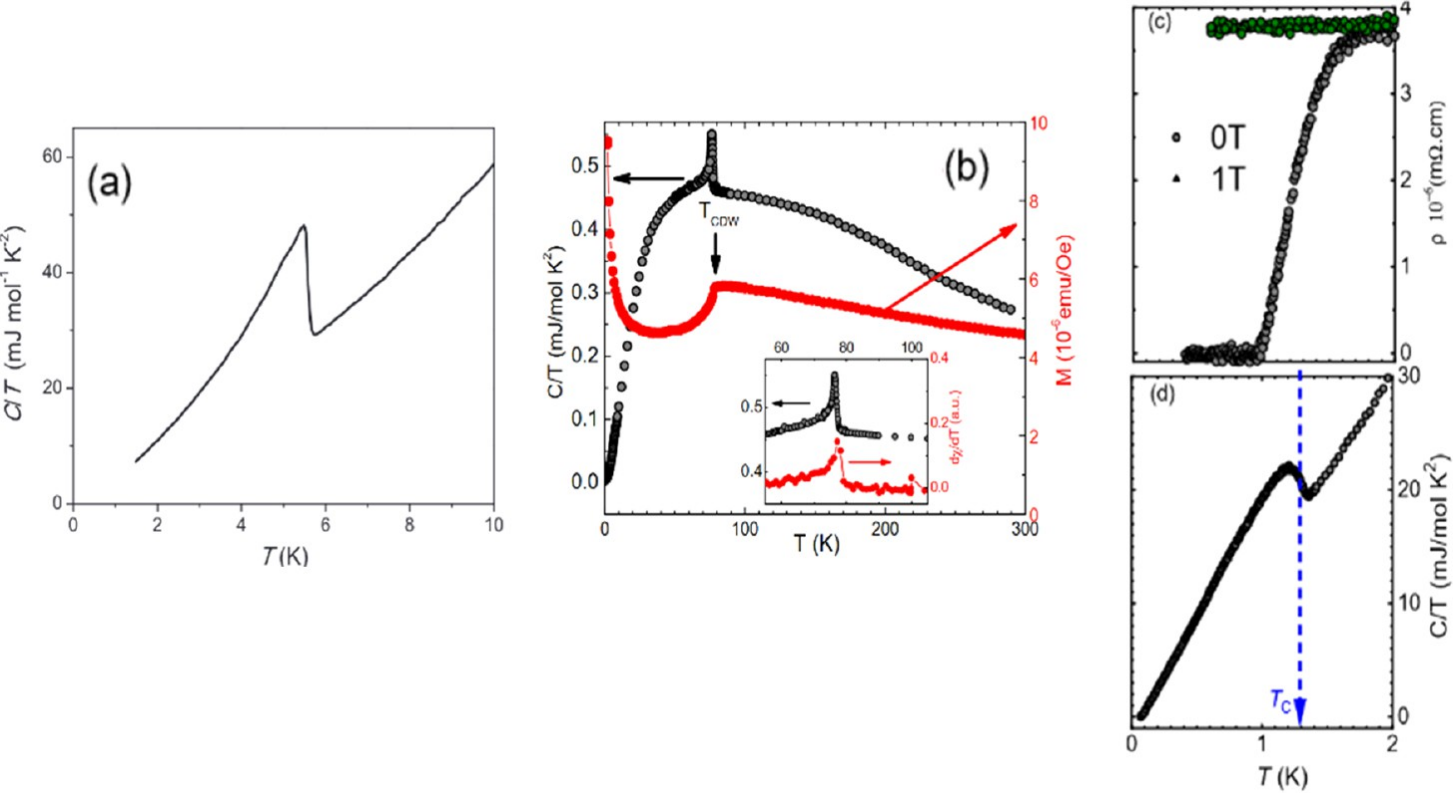
Thermodynamic properties. (a) Zero-field specific heat *C*/*T* of the NbS_2_ single crystal. A sharp superconducting transition is centered at *T*_c_ = 5.5 K. (b) Temperature dependence of the magnetic susceptibility and specific heat 2H-TaS_2_. The right side presents the *T*-dependence of magnetization measured under 0.5 T parallel to the crystallographic basal plane. The left side shows the specific heat data measured in zero-field conditions. The inset shows the enlarged *C*_p_/*T* vs *T* plot and the derivative of the magnetization near the CDW transition. (c and d) Temperature dependence of resistivity at zero and 1T magnetic fields and specific heat at zero fields, respectively.

The synthetic routes reported here produce two-dimensional transition metal dichalcogenides (TMDCs) that can form charge density waves (CDWs), be superconducting or magnetic, gapped or metallic, and overall with a range of physical properties that reflect the underlying electronic structure^16−19^ (Figures S3 and 7). Bulk superconductivity in NbS_2_ is illustrated from low-temperature specific heat measurements, as shown in Figure 7a. Remarkably, we find that superconductivity beyond the Pauli limit still exists in bulk single crystals of NbS_2_ for a precisely parallel field alignment, and our upper critical field points to the development of a Fulde–Ferrell–Larkin–Ovchinnikov state above the Pauli limit as the main mechanism. This is also consistent with the observation of a magnetic field-driven phase transition in the thermodynamic quantities within the superconducting state near the Pauli limit. In 2H-TaS_2_, the temperature dependence of specific heat and magnetization measurements are illustrated in Figure 7b. A clear maximum of both data at 76 K is an indication of the charge density wave (CDW) transition. Below the CDW transition, the magnetic susceptibility decreases sharply and continuously. This decrease in the susceptibility can be attributed to the decrease in the density of states on the hole band due to the opening of the CDW gap, and additionally, no long-range magnetic order was detected in this material. Bulk superconductivity in TaS_2_ is illustrated from low-temperature resistivity and specific heat measurements, see Figure 7c,d. To make a complete report of the properties of the here synthesized compounds, we discuss in the Supporting Information (SI) the calculated electronic structure of several transition metal dichalcogenides (i.e., CoS_2_, ReS_2_, NbS_2_, and TaS_2_). These systems were selected to provide insight into the interdependence of the crystal structure, chemical composition, and electronic structure (Figures S1 and S2).

In summary, we have demonstrated that sulfur vapors with a fugacity of approximately 1–5 atmospheres allow us to transport many transition metals and to obtain crystals of sulfides with the maximum possible sulfur content for these conditions. In the absence of sulfur, the transport of these elements was impeded. Normally, crystals grew closer to the cooler part of the ampoule next to liquid sulfur. In several experiments, transported metals partly dissolved in liquid sulfur and crystallized as sulfides right therein. Our synthetic routes reported here open for the design and growth of high-quality single crystals for TMDC research and applications and will allow an unprecedented characterization of the physical properties, which leads to a better understanding of the underlying mechanisms of TMDC research.

## Methods Summary

### Chemical Composition

The chemical composition of the crystals obtained was measured using a Tescan Vega II XMU scanning electron microscope with an INCA Energy 450 energy-dispersive spectrometer at an accelerating voltage of 20 kV. Crystals glued to the conducting substrate as well as embedded into polished epoxy resin were studied. The crystals were ground and examined by an X-ray powder diffraction technique on DRON-7 (Co K_α_-radiation) or BRUKER (Cu K_α1_-radiation, graphite monochromator) diffractometers. Crystals with an apparent layered structure were checked for monocrystallinity on the BRUKER diffractometer. Traces of transport were found almost in all ampoules after 2–4 months in the furnace when the temperature in the hot end was 850–820 and 570–540 °C in the cooler end and if sulfur excess was sufficient. Most often crystals were found closer to the cooler end of the ampoule, that is, the temperature of crystallization was not greater than 650 °C.

### Magnetization Measurements

Magnetization measurements were performed by using a Quantum Design SC quantum interference magnetometer. The low-T specific heat down to 0.4 K was measured for TaS_2_ in its physical property measurement system with the adiabatic thermal relaxation technique. Specific heat measurements were performed down to 70 mK by using a heat-pulse technique within a dilution refrigerator along *H*||*c*.

### Theory

We have performed spin-polarized plane-wave pseudopotential calculations with norm-conserved projector-augmented wave (PAW) pseudopotentials, as implemented in the Vienna Ab initio Simulation Package (VASP).^20^ The exchange–correlation interactions are treated under generalized gradient approximation (GGA) with van der Waals corrections according to the Grimme DFT-D2 method.^21^ The cutoff energy for the plane-wave expansion is set as 500 eV.

## Supporting Information Available

Any additional methods, extended data display items, and source data are available in the online version of the paper; references unique to these sections appear only in the online paper (PDF)
