# Correction to Stable Sulfuric Vapor Transport and Liquid-Sulfur Growth on Transition Metal Dichalcogenides

**DOI:** 10.1021/acs.cgd.3c00436

**Published:** 2023-05-08

**Authors:** Dmitriy A. Chareev, Md Ezaz Hasan Khan, Debjani Karmakar, Aleksey N. Nekrasov, Maximilian S. Nickolsky, Olle Eriksson, Anna Delin, Alexander N. Vasiliev, Mahmoud Abdel-Hafiez

Details of the correction only to the Acknowledgments:

