# Supplementary material for: Stable Sulfuric Vapor Transport and Liquid Sulfur Growth on Transition Metal Dichalcogenides
Source: Cryst Growth Des. 2023 Mar 21;23(4):2287–94. doi: 10.1021/acs.cgd.2c01318 (PMC10080655; doi:10.1021/acs.cgd.2c01318)
Supplement: Supplementary file 1 — cg2c01318_si_001.pdf [file cg2c01318_si_001.pdf]

Supplementary Information for

Stable Sulfuric Vapor Transport and Liquid-Sulfur Growth on Transition Metal Dichalcogenides

Dmitriy A. Chareev*[1,2,3], Md Ezaz Hasan Khan[4], Debjani Karmakar[5], Aleksey N. Nekrasov[1], Maximilian S. Nickolsky[6], Olle Eriksson[5,7], Anna Delin[8,9], Alexander N. Vasiliev[10,11], Mahmoud Abdel-Hafiez*[5,4]

[1] Institute of Experimental Mineralogy (IEM RAS), 142432 Chernogolovka, Moscow Region, Russia
[2]Kazan Federal University, 18 Kremlyovskaya St., 420008 Kazan, Russia
[3]Ural Federal University, Ekaterinburg 620002, Russia
[4]University of Doha for Science and Technology, Doha, P.O. Box 24449, Qatar
[5] Department of Physics and Astronomy, Uppsala University, Box 516, SE-75120 Uppsala, Sweden
[6]Institute of Geology of Ore Deposits (IGEM RAS), 35, Staromonetnyi per., 119017 Moscow, Russia
[7]School of Science and Technology, Örebro University, SE-701 82 Örebro, Sweden
[8]Department of Applied Physics, KTH Royal Institute of Technology, SE-106 91 Stockholm, Sweden
[9]Swedish e-Science Research Center, KTH Royal Institute of Technology, SE-10044 Stockholm, Sweden
[10]Lomonosov Moscow State University, Moscow 119991, Russia
[11]National University of Science and Technology "MISiS", Moscow 119049, Russia

I. <u>Theoretical studies on Sulfides</u>:

The analysis of the correlation of structural and optical properties of the bulk sulfide single crystals *viz.* CoS_2, ReS_2, TaS_2 and NbS_2 can be accomplished by exploring their electronic structures with the help of the first-principles-based density-functional investigation. The Monkhorst-pack grid used for the Brillouin zone sampling of the cubic system is $5 \times 5 \times 5$ and for all non-cubic systems, we have used the sampling of $5 \times 5 \times 3$. The ionic positions and the lattice parameters are relaxed within the framework of conjugate gradient algorithm until the Hellmann-Feynman force on each ion is less than 0.01 eV/ Å.

Whereas the ground-state crystal structure of bulk CoS_2 belongs to the cubic pyrite-like structure with the space group la-3, bulk ReS_2 pertains to a monoclinic structure with space group P1 [1]. The structural representations of the unit cells of CoS_2 and ReS_2 are depicted in Fig. SI 1(a) and (b) with the

corresponding number of formula units per unit cell being four and eight respectively. The corresponding orbital projected band-structures are presented in Fig.S1(c) and (d), which reveal that within GGA, CoS_2 is a ferromagnetic metal and ReS_2 is a direct band-gap semiconductor with negligible magnetic moment. In the ground-state magnetic configuration, the four different Co ions in CoS_2 areferromagnetically aligned in a low-spin electronic configuration, having an average magnetic moment of 1.15 μ_B per Co-ion. Near the Fermi-level, the S-3*p* states are strongly hybridized with partially-filled Co-e_g levels in an octahedral crystal-field environment and the t_{2g}-levels of Co-3*d* states are fully filled in an energy range of -3 to -1.5 eV, as from the Fig.S1(c). On the other hand, ReS_2 has a strong orbital anisotropy, leading to different contributions from the in-plane and out-of-plane orbitals at the valence band maxima (VBM) and conduction band minima(CBM). There is a direct band-gap of ~ 1.25 eV at the Γ-point. States at the VBM are dominated by the highly hybridized in-plane Re 5*d*-orbitals (d_{x2-y2} and d_{xy}) and S-3*p* planar orbitals (p_x and p_y). The states at the CBM have more contributions from the out of plane d_{xz}, d_{yz}, d_{3z2-1} orbitals hybridized with the S-3p_z orbitals.

The next two systems, TaS_2 and NbS_2 are well-known charge-density wave (CDW) compounds. For both of them, the most commonly occurring phase is hexagonal, belonging to the space group P63/mmc with two formula units per unit cell. Figs. SI 2(a) and 2(b) depict the structural representation of these two compounds. For both of these compounds the chemical interaction along the c-axis is via van der Waal forces. The orbital-projected band structures for these two compounds are presented in Figs. SI 2(c) and 2(d), revealing metallic behaviour, where the Fermi-level is populated by partially filled S-3*p*, Ta-5*d* and Nb-4*d* levels. The crystal-field coordination is trigonal prismatic, with strong in-plane sigma-bonding between the Ta (5*d*) and Nb (4*d*) d_{xz}, d_{yz}, d_{x2-y2} and d_{xy}orbitals, and the S-3p_x and 3p_y orbitals, which constitutes are bonding levels. At the Fermi-level, there are electron and hole pockets from S-3*p*, Ta-5*d* and Nb-4*d* states, which indicates the possible presence of CDW.

In summary, the electronic structure of these transition-metal dichalcogenides provide an insight about the interdependence of the chemical composition, crystal structure and their response to the photoemission and x-ray absorption measurements.

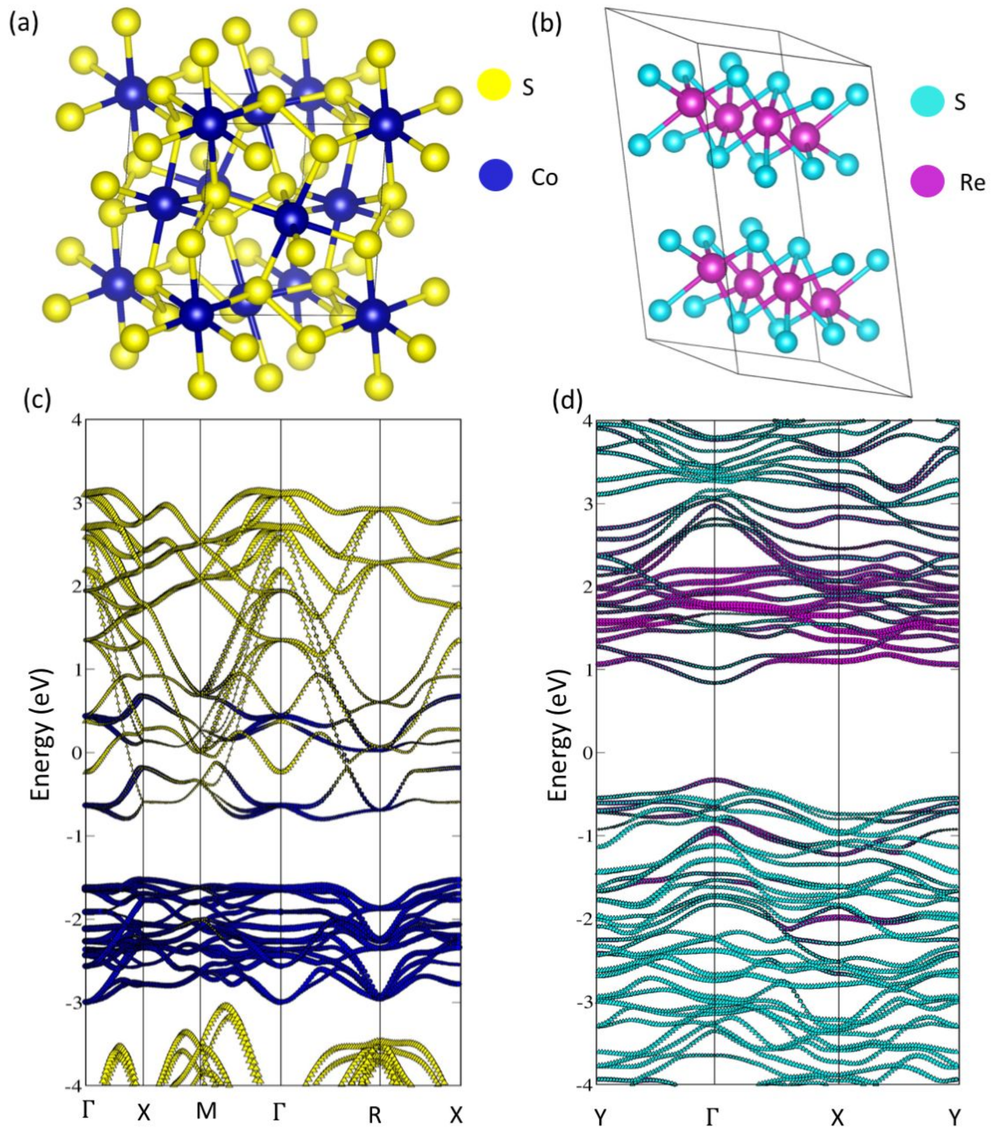

Figure SI. 1|Theoretical studies on CoS_2 and ReS_2: (color online) (a) The unit cell of CoS_2 in the cubic Pyrite structure, (b) The monoclinic unit cell of ReS_2, (c) GGA orbital projected band-structure of CoS_2, the blue and the yellow color designate the Co-3*d* and S-3*p* orbital character, (d) GGA orbital projected band-structure of ReS_2, the magenta and the cyan color designate the Re-5*d* and S-3*p* orbital characters.

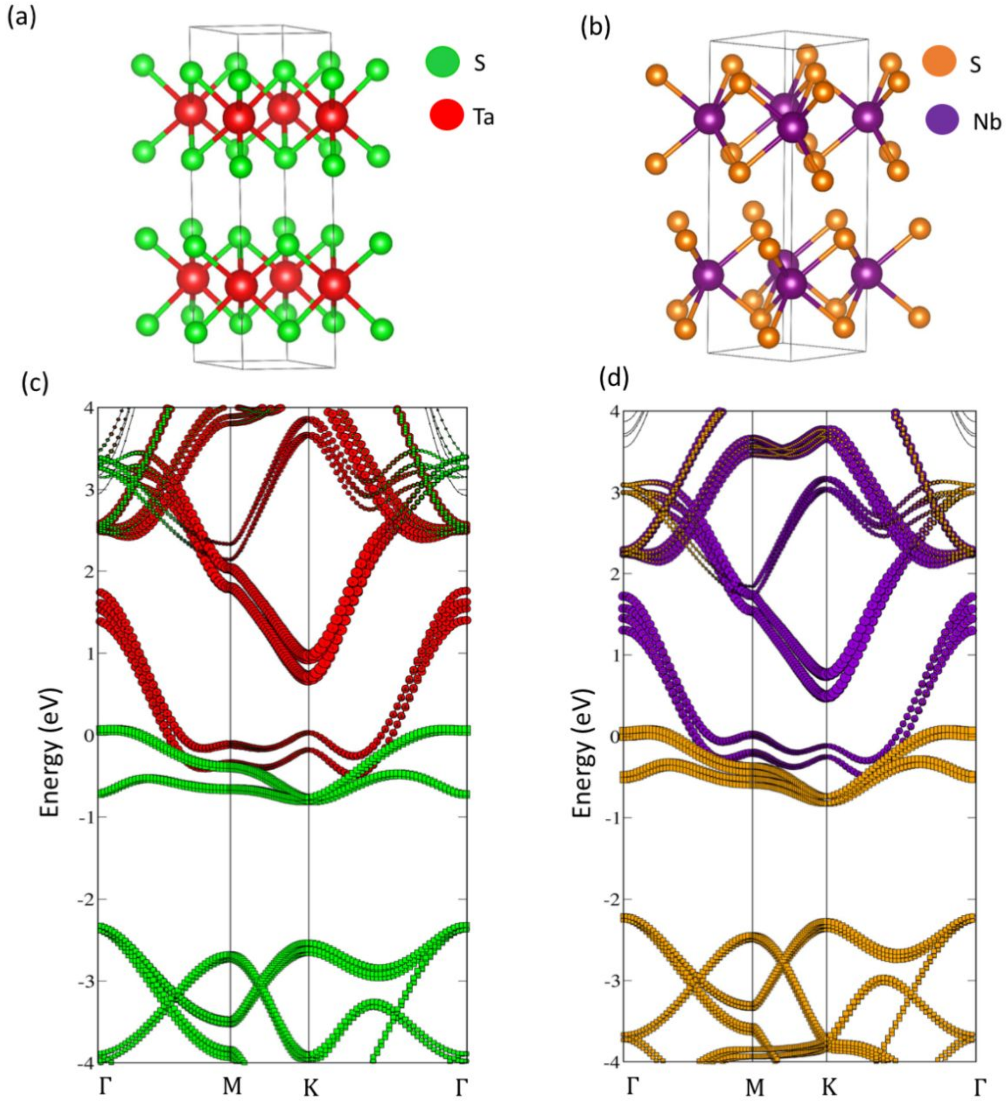

Figure SI. 2|Theoretical studies of NbS_2 and TaS_2(colour online) (a) The unit cell of TaS_2 in the hexagonal structure, (b) The unit cell of NbS_2 in the hexagonal structure, (c) GGA orbital projected band-structure of TaS_2, the red and the green colours designate the Ta-5*d* and S-3*p* orbital character, (d) GGA orbital projected band-structure of NbS_2, the violet and the orange colours designate the Nb-4*d* and S-3*p* orbital characters.

II. Magnetic Thermodynamic properties

In Fig. SI. 3 (left), we present the temperature dependence of the magnetization for CoS_2. The magnetization increases with decreasing temperature, with a Curie temperature at 120 K. Figure 6 (right) shows the magnetic field dependence below and above the Curie temperature. These results agree also with reports in the literature [18-22] main text.

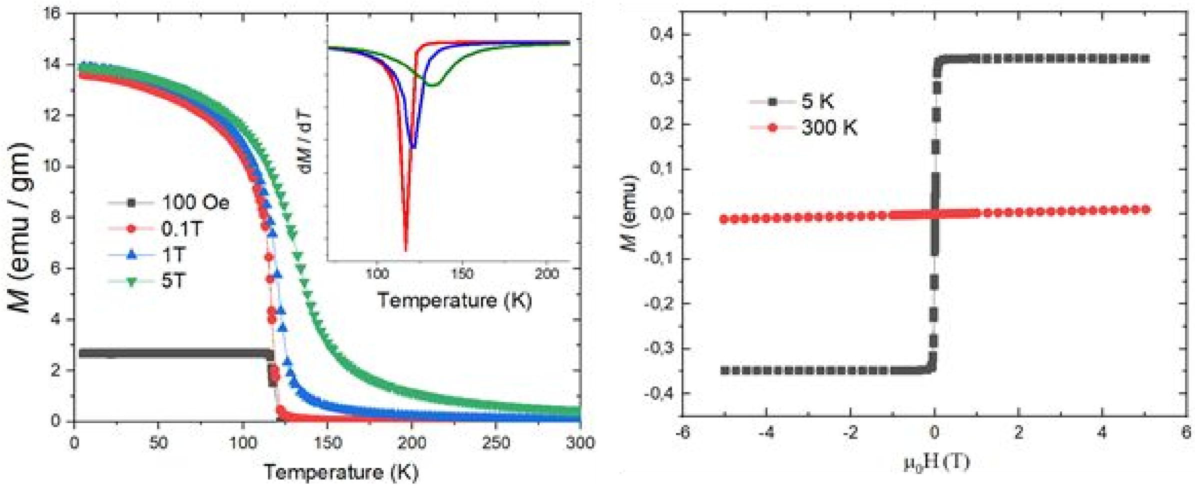

Figure SI. 3|Magnetic properties. (*left*) illustrates the temperature dependence of magnetization at various applied external fields for CoS_2. inset shows the corresponding d*M*/dT versus T curves and (*right*) presents the field dependence of magnetization at 300K and 5K for CoS_2. The measurements were taken along the [100] direction.

References:

[1] Gadde, R.et al. Two-dimensional ReS_2: Solution to the unresolved queries on its structure and inter-layer coupling leading to potential optical applications. Phys. Rev. Materials 5, 054006 (2021). https://doi.org/10.1103/PhysRevMaterials.5.054006
